# Changes in Soil Bacterial Community and Function in Winter Following Long-Term Nitrogen (N) Deposition in Wetland Soil in Sanjiang Plain, China

**DOI:** 10.3390/microorganisms11112634

**Published:** 2023-10-26

**Authors:** Rongtao Zhang, Xiaoyu Fu, Haixiu Zhong, Xin Sui, Yingnan Liu

**Affiliations:** 1 Institution of Nature and Ecology, Heilongjiang Academy of Sciences, Harbin 150040, China; zhangrongtao14@163.com (R.Z.); 18646583130@163.com (X.F.); zhx971030@163.com (H.Z.); 2 Heilongjiang Provincial Key Laboratory of Ecological Restoration and Resource Utilization for Cold Region, School of Life Sciences, Heilongjiang University, Harbin 150040, China

**Keywords:** N deposition, winter, soil bacterial, Sanjiang plain

## Abstract

N deposition is a key factor affecting the composition and function of soil microbial communities in wetland ecosystems. Previous studies mainly focused on the effects of N deposition in the soil during the growing season (summer and autumn). Here, we focused on the response of the soil microbial community structure and function in winter. Soil from the Sanjiang Plain wetland, China, that had been treated for the past 11 years by using artificial N deposition at three levels (no intervention in N0, N deposition with 4 g N m^−2^ yr^−1^ in N1, and with 8 g N m^−2^ yr^−1^ in N2). Soil characteristics were determined and the bacterial composition and function was characterized using high-throughput sequence technology. The N deposition significantly reduced the soil bacterial diversity detected in winter compared with the control N0, and it significantly changed the composition of the bacterial community. At the phylum level, the high N deposition (N2) increased the relative abundance of Acidobacteria and decreased that of Myxococcota and Gemmatimonadota compared with N0. In soil from N2, the relative abundance of the general *Candidatus_Solibacter* and *Bryobacter* was significantly increased compared with N0. Soil pH, soil organic carbon (SOC), and total nitrogen (TN) were the key factors affecting the soil bacterial diversity and composition in winter. Soil pH was correlated with soil carbon cycling, probably due to its significant correlation with aerobic_chemoheterotrophy. The results show that a long-term N deposition reduces soil nutrients in winter wetlands and decreases soil bacterial diversity, resulting in a negative impact on the Sanjiang plain wetland. This study contributes to a better understanding of the winter responses of soil microbial community composition and function to the N deposition in temperate wetland ecosystems.

## 1. Introduction

The combustion of fossil fuels and certain agricultural practices have led to a significant increase in reactive nitrogen input to terrestrial ecosystems. It is estimated that anthropogenic nitrogen input now exceeds the amount of reactive nitrogen produced via biological nitrogen fixation by about 210 Tg.a^−1^ [1]. Continuous local N deposition eventually leads to excess nitrogen in the soil, which ultimately changes soil ecosystems and their structure and function [2,3], including soil microbial composition and diversity [4,5].

Soil bacteria play crucial roles, including the regulation of many ecological functions, litter decomposition, and biogeochemical element cycling, thereby ensuring and maintaining the biodiversity of plants and animals [6]. With their sensitivity to environmental changes, bacteria serve as important indicators to assess soil nutrient cycling and ecosystem stability [7,8]. The deposition of N can influence the structure and function of the soil microbiome directly, by increasing the N availability [9,10], and indirectly, by decreasing the soil pH and SOC [11]. On one hand, certain microorganisms that depend on inorganic nitrogen as their energy source can increase along with the N deposition, potentially increasing the microbial diversity. On the other hand, oligotrophic microorganisms can decrease in numbers as a result of higher N availability [6,12], and this results in a decline of microbial diversity. Moreover, the N deposition can indirectly affect soil microbial functions, compositions, and diversities via changes in the above-ground vegetation composition and soil properties [13,14]. For example, Lu et al. [15] reported that soil bacteria varied in abundance as a result of N deposition treatments in the coastal wetlands of the Yellow River Delta, with higher N concentrations leading to increased nutrients in the soil combined with a decreased soil microbial diversity. However, for the surface layer of Qinghai Lake wetlands, it was found that the N addition increased the soil bacterial diversity and that the community composition was mainly influenced by soil physicochemical factors [14]. Another study showed that the N deposition affected the functional groups of microorganisms involved in the soil carbon and nitrogen cycle, and while it altered the structure and interactions of soil microbial communities, the effects of different forms and levels of N deposition were not consistent [16]. Although the effects of the N addition on the structure and function of wetland soil bacterial communities have been extensively investigated during the growing season, there is a considerable paucity of studies on the response of soil communities to the nitrogen addition in winter.

In general, biological activity is weak or even ceases when temperatures fall below freezing, a period known as the dormant season [17]. However, soil microorganisms remain active even when the soil is frozen and they still contribute to biogeochemical cycling processes during winter [18,19]. In fact, that season may be an important period affecting many ecological processes. The response of soil microbial communities and its diversity to the N deposition varies between the seasons, although it depends on the local climate. In subtropical forests, it was observed that the N addition increased the soil bacterial diversity both in winter and in summer [20], but in temperate forests, the N deposition mostly decreased in bacterial abundance and diversity in winter [21]. The different responses of the soil microbial structure and diversity to the N deposition in ecosystems under different climatic conditions has mostly been studied for forest ecosystems, while the winter responses of wetland ecosystems is far less intensely researched.

Wetlands represent one of the most important terrestrial ecosystems in terms of high biodiversity, and although they only cover 4–6% of the Earth’s surface area, they play an important role in biogeochemical cycles [22,23]. The Sanjiang Plain is a large, continuous, well-maintained fresh wetland situated in the northeast of China. This area is an important grain production base, so it is of high importance both for ecological reasons and for food security. The structure and function of soil microorganisms of the Sanjiang plain wetland ecosystem has been seriously affected by exogenous nitrogen inputs, resulting in declined biodiversity and changes in biogeochemical cycling processes [12]. Observations collected in summer suggest that the effect of the N addition to the soil bacterial diversity was not severe, but it decreased the fungal diversity; therefore, how the soil microorganisms respond in winter remains to be determined.

A long-term experiment was started in May 2010, where on a yearly basis, N was deposited at two different concentrations onto a typical wetland in the Sanjiang plain, with a third plot receiving no intervention serving as a control. We have studied the soil microbial diversity and composition after a short-term N deposition (4 year) in summer and found that a short-term N deposition did not change the soil bacterial diversity but significantly affected the soil fungal diversity (Sui et al., unpublished). However, why do soil microorganisms not respond to the long-term N deposition? Therefore, after ten consecutive years (2020), the soil was sampled in winter and analyzed for soil bacterial diversity and composition using high-throughput sequencing technology. 

The aim of this study was to investigate in what way the continuous N deposition at two concentrations had affected the diversity and composition of the soil bacterial community’s detectability in winter and to identify the relationships between the soil bacteria and soil properties under the studied conditions, in order to contribute to a deeper understanding of the responses of wetland ecosystems in Sanjiang plain to the increasing N depositions.

## 2. Materials and Method

### 2.1. Description of the Study Area

**Figure 1:**
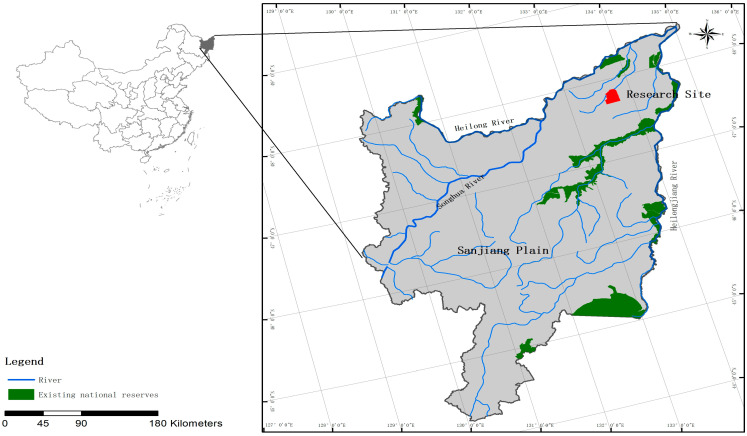
Map of the research site in the Sanjiang Plain, China.

This study was performed at a field experimental station of Heilongjiang Academy of Sciences, in the Honghe National Nature Reserve (47°42′18″–47°52′07″ N, 133°34′38″–133°46′29″ E), Heilongjiang Province, China (Figure 1). The climate is a typical temperate humid/semi-humid monsoon climate, with an average annual temperature of 1.9 °C and an average annual precipitation of 585 mm with 1166 mm evaporation [10]. The local vegetation is dominated by *Deyeuxia angustifolia*, *Glyceria spiculosa*, *Carex lasiocarpa*, and *Carex pseudocuraica*. The main soil types are meadow and peat marsh soil (Gray Luvisols, FAO soil classification).

### 2.2. Experimental Design

The N deposition experiment was set up in the peat marsh *Deyeuxia angustifolia* wetland. The N deposition experiment, which ran from 2010 to 2020, involved an area in which nine 10 m × 10 m plots were established, separated by at least 1 m. The setup was previously described [12] and involved the deposition of N fertilization in the form of dissolved ammonium nitrate that was sprayed once a month from May to October. Treatment was compared between a low nitrogen load in N1 applying in a total of 4 g N m^−2^ per year and a high nitrogen load in N2 with 8 g N m^−2^ per year. Control plots N0 received water only. All treatments were performed in triplicates. 

### 2.3. Soil Sampling

In February 2021, after 10 years of N deposition as described above, soil samples were collected from each treatment plot following a standard protocol previously described [12]. Samples were taken at 0~10 cm depth (after removal of the 0–20 cm litter layer) with an auger with a 5 cm diameter. From each plot, 10 samples of 2.5 kg soil were randomly taken, mixed, and after the removal of roots and stones, they were transported to the laboratory at 4 °C. The soil was passed through a 2 mm mesh and 10 g was stored at −80 °C for DNA extraction, while approximately 1.5 kg was air dried for soil physicochemical analyses.

### 2.4. Soil Chemical Properties

The chemical properties of pH, TN, SOC, available nitrogen (AN), and total phosphorus (TP) of the soil were determined as described by Fu et al. [24]. For determining the pH, a soil–water suspension (1:2.5 *w*/*v*) was first shaken for 30 min. TN and SOC were determined using an Elemental Analyzer (Elementar, Langenselbold, Germany). To determine the AN content, the soil was sequentially acid digested and AN was determined with a continuous flow analysis system (SKALAR SAN++, Breda, The Netherlands). TP was determined spectrophotometrically after acid digestion and AP was measured via colorimetry after the NaHCO_3_ extraction, as previously described [24]. 

### 2.5. DNA Extraction, PCR Amplification, and MiSeq Sequencing

The total DNA present in the soil was extracted using the QIAGEN DNeasy PowerSoil Pro Kit (Omega Bio-Tek, Norcross, GA, USA) and this was used for amplification of the V3–V4 region of the bacterial 16S rRNA gene using universal primers 338F and 806R, as described in the literature [24]. Each PCR product was amplified in three replicates and mixed together into one PCR product. Each N treatment includes three PCR replicates. Following purification, the amplicons were sequenced by Biomaker Biotechnology Co., Ltd (Beijing, China). 

Processing of the raw sequences was performed using QIIME1 and all subsequent steps of the data processing are described in Fu et al. [24]. Briefly, forward and reverse reads were merged using PEAR software (v.0.9.8). Sequences were removed if the mean quality score was <20 or if the length was <200 bp, and ambiguous sequences were also removed. Chimers were moved using Usearch (v7.1, http://drive5.com/usearch/ accessed on 2 March 2023). Exact barcode matching was implemented, which permitted a two-nucleotide mismatch during primer matching. A similarity of 97% was applied to sort reads into operational taxonomic units (OTU) via BLAST against the SILVA database (v138.1, https://www.arb-silva, accessed on 2 March 2023). All reads were normalized according to the lowest numbers of a sample before further analysis. 

### 2.6. Statistical Analyses

The online platform of Majorbio Cloud Platform (www.majorbio.com) was used for the data analysis. The α diversity indices for Chao1 and Shannon was computed via QIIME1. For statistical analysis, non-metric multidimensional scaling (NMDS) and permutational Multivariate Analysis of Variance (PERMANOVA) was based on the Bray–Curtis dissimilarity at the OTU level and Venn diagrams were generated at the OTU level. 

Online tools (https://www.bioincloud.tech/, accessed on 2 March 2023) were used for the following analyses: cladograms and LEfSe were generated for phyla and genera; heatmaps were based on the 30 most abundant bacterial genera; correlation heatmaps included soil physicochemical parameters and the bacterial α index; and redundancy analysis (RDA) correlated the soil physicochemical parameters with bacterial OTUs. 

Based on OTUs, the FAPROTAX functions of bacteria were predicted as previously described [25]. The significance of any determined differences was determined via Duncan’s multiple comparison method of one-way analysis of variance (ANOVA) with a detection level of 0.05. The one-way ANOVA of FAPROTAX in the relative abundance was calculated at a 0.05 significance level. All statistical analyses were conducted using SPSS 23.0 software.

## 3. Results

### 3.1. Changes of Soil Physico-Chemical Properties under N Deposition

The soil properties determined for the samples collected in winter varied depending on the N depositions that had been applied during the previous 10 years (Table 1). Compared to the control N0, which had not received any nitrogen, the soil of N1 that had been dosed with a low concentration of N had significantly decreased the SOC and TN concentrations (*p* < 0.05). There was no difference for the soil pH and for the AN, TP, and AP concentrations between N1 and N0 (Table 1, *p* > 0.05). Soil from N2 that had received the high deposit of N had a significantly lower pH and lower levels of TP, AP, SOC, and TN (*p* < 0.05, Table 1).

### 3.2. N Deposition Changes the Soil Bacterial α and β Diversity

**Figure 2:**
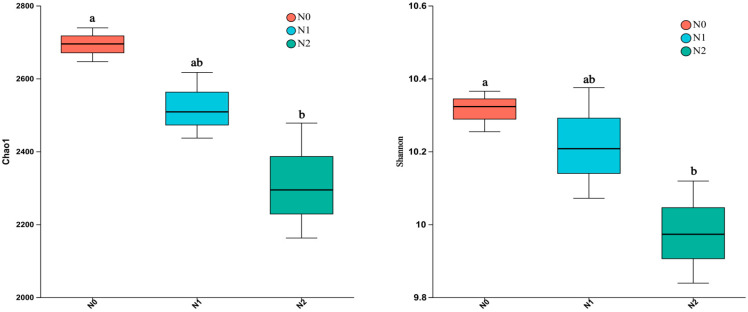
Bacterial α diversity (Chao1 to the left and Shannon to the right) in the soil under the three different treatments. Treatment N0: no N applied; treatment N1: 40 kg N·hm^−2^ per year for 10 years; and Treatment N2: 80 kg N·hm^−2^ per year for 10 years. Each box includes three replicates. Different letters within a row indicate significant differences (*p* < 0.05; ANOVA) among the three different treatments.

As the soil bacterial communities were studied via 16S rRNA amplicon sequencing and the α diversity of the identified populations was calculated using the Shannon and Chao1 indices. As shown in Figure 2, these indices were highest for the soil bacteria detected in N0 and lowest in N2. The difference between N0 and N2 was significant (*p* < 0.05; Figure 2), but between N0 and N1, it was not (*p* > 0.05).

### 3.3. N Deposition Change the Bacterial Compositions

**Figure 3:**
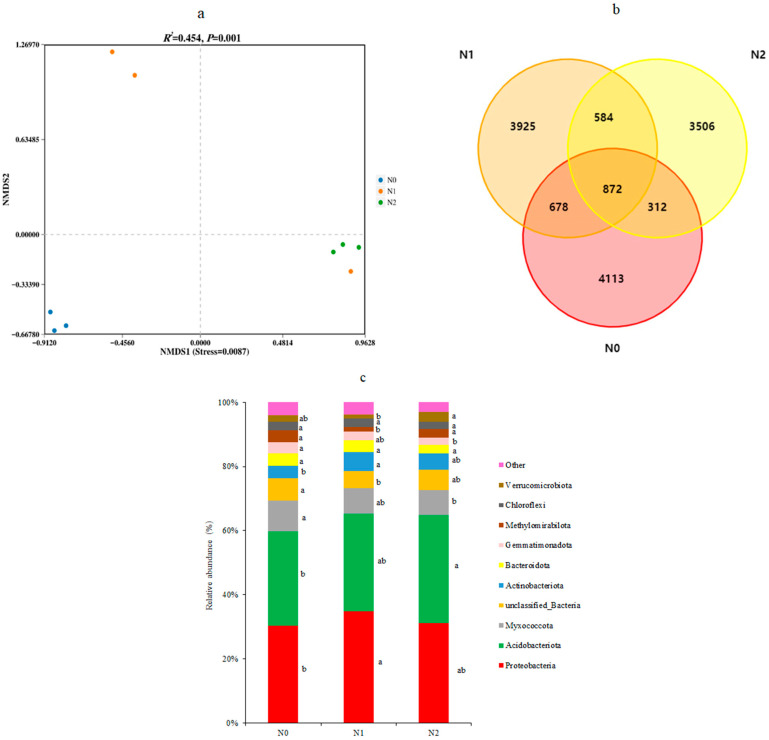
The soil bacterial structure and composition of three N deposition in Sanjiang plain wetland. (**a**) Indicated NMDS plot of the soil bacterial OTUs based on Bray Curtis metrics among all samples; (**b**) indicated Venn diagram of the number of OTUs identified in the soil under different nitrogen deposition treatments N0, N1, and N2; and (**c**) indicated relative abundance of the bacterial phyla in the three soils. N0, 0 kg N·hm^−2^·a^−1^; N1, 40 kg N·hm^−2^·a^−1^; and N2, 80 kg N·hm^−2^·a^−1^. Different letters within a row indicate significant differences (*p* < 0.05; ANOVA) among the three different treatments in (**c**).

NMDS based on confidence intervals (*p* < 0.01) was performed and this showed an absence of overlap between the different treatments (Figure 3a). This illustrated that the soil bacterial communities (stress = 0.0087) were clearly different in the soils of the three different treatments, with the possible exception of one N1 sample, which was grouped with the three N2 samples. PERMANOVA analysis confirmed that the different treatments significantly altered the bacterial community’s β diversity (significant difference between groups, R2 = 0.454, *p* < 0.01).

The effects of N deposition on soil bacterial composition are shown in Figure 3. The number of OTUs identified in the soil was similar between N0 and N1 (5975 and 6059, respectively), but lower in N2 (5274). The total number of OTUs that were detected was 13,990. There were 872 OTUs that were found shared among the soils of the three treatments (Figure 3b), representing approximately 6% of the total OTUs. The largest number of unique OTUs was identified in N0 soil (4113) and these represented 68.8% of all OTUs from that treatment. For comparison, N1 contained 3925 unique OTUs (64.8% of its total OTUs) and N2 had 3506 unique OTUs (66.7%). 

The most abundant bacterial phyla were Proteobacteria and Acidobacteria, which in combination, reached between a 60% (N0) and 68% (N1) abundance, followed by Myxococcota and Actinobacteriota (Figure 3c). The relative abundance (r.a.) of Proteobacteria, Acidobacteria, Myxococcota, Actinobacteriota, and other dominant bacterial phyla were significantly different between the three N deposition treatments (*p* < 0.05). Compared to the control N0, a decade of low-level N deposition in N1 had significantly increased the r.a. of Proteobacteria and Actinobacteriota, while that of a number of less abundant phyla had also changed. Comparing N0 with the high-level N deposit in N2 revealed a significant increase in the r.a. of Acidobacteria and a decrease in Myxococcota and Gemmatimonadota (Figure 3c).

**Figure 4:**
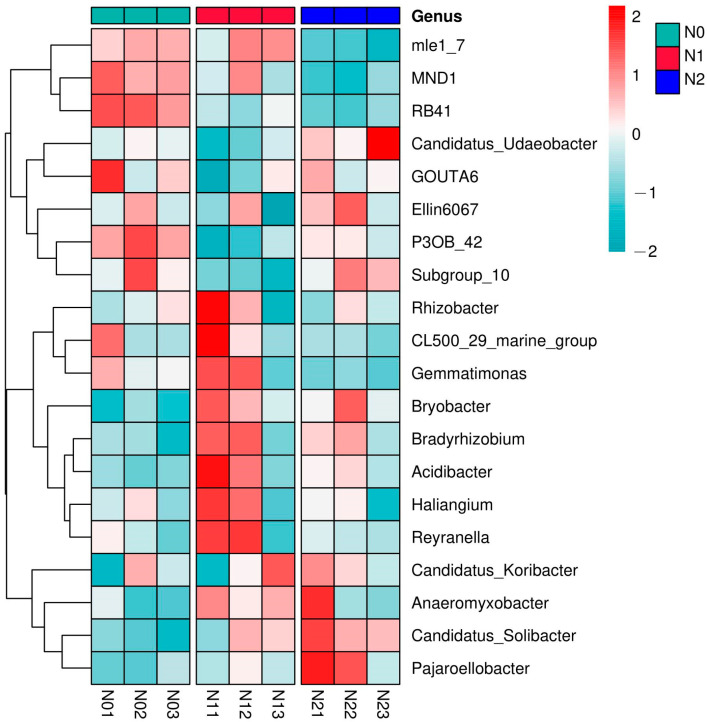
Heatmap of the top 20 most abundant bacterial genera in the different nitrogen deposition treatments. Triplicates are shown of N0 (N01, N02, N03), of N1 (N11, N12, N13), and of N2 (N21, N22, N23). N0, 0 kg N·hm^−2^·a^−1^; N1, 40 kg N·hm^−2^·a^−1^; and N2, 80 kg N·hm^−2^·a^−1^.

To compare the shifts in relative abundance at the genus level, a heatmap was generated for the 20 most abundant bacterial genera for all triplicate soil samples (Figure 4). This identified the co-enrichment patterns between genera, as indicated by the genera mle_7, MND1, and RB41 (forming the top cluster in the figure), which were co-enriched in all N0 samples compared to N2, or the four genera grouping in the bottom cluster that were enriched in N2 compared to N0. The highest enrichment in the N0 soil samples was observed for the members of the top two clusters (from mle1_7 to Subgroup_10). In N1, the genera grouped in the middle two clusters were overabundant (from Rhizobacter to Reyranella), and the lower cluster (from Candidatus_Koribacter to Pajaroellobacter) was dominant in N2 (Figure 4). 

The absolute abundance of genera with marked differences between the treatments when the triplicates were averaged is summarized in Table 2. The top 10 genera are sorted for decreasing abundance in N0. The decade-long deposition of N consistently increased the absolute abundance of Candidatus_Solibacter in a concentration-dependent manner, and Bryobacter also increased as a result of N deposition while that treatment decreased the relative abundance of P3OB_42 (in particular in N1) and of MND1 in N2 (*p* < 0.05). The abundance of Candidatus_Udaeobacter was significantly higher in N2 compared to N1 (*p* < 005).

**Figure 5:**
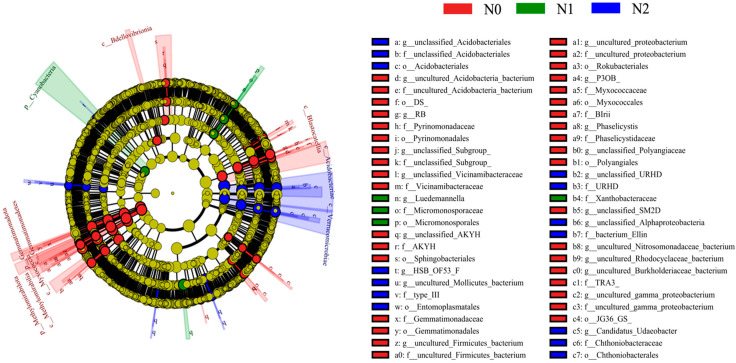
Cladogram of the soil bacterial communities in the soil following different nitrogen deposition treatments with liner discriminant analysis (LDA) > 2.5. The circles represent bacterial taxa from phyla (center) to genera (outer ring). N0, 0 kg N·hm^−2^·a^−1^; N1, 40 kg N·hm^−2^·a^−1^; and N2, 80 kg N·hm^−2^·a^−1^.

A linear discriminant analysis effect size (LEfSe) was performed to identify which bacterial taxa had changed in abundance that could most likely explain the differences between treatments. This identified 54 taxa for which significant differences were recorded in the three treatments, as indicated by LDA effect size scores higher than 2.5 (Figure 5). At the genus level, RB41, P3OB_42, and Phaselicystis were indicator genera for N0; Luedemannella was an indicator genus for N1; and Candidatus_Udaeobacter and HSB_OF53_F07 were indicator genera for N2.

### 3.4. Soil Bacterial Functional Groups

**Figure 6:**
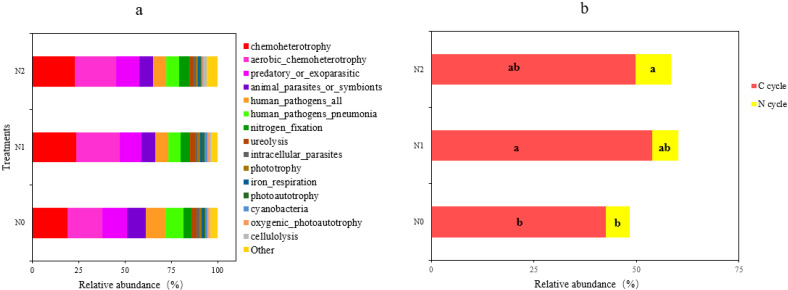
Soil bacterial functional profile (**a**) and functional group composition (**b**) related to the carbon cycle (in red) and the nitrogen cycle (in yellow) for the three treatments. N0, 0 kg N·hm^−2^·a^−1^; N1, 40 kg N·hm^−2^·a^−1^; and N2, 80 kg N·hm^−2^·a^−1^. Different letters within a row indicate significant differences (*p* < 0.05; ANOVA) among the three different treatments in (**b**).

The functional capacity of the detected bacteria was predicted using the FAPROTAX database, from which we obtained a total of 51 functional groups. The 15 most abundant functional groups with an r.a. > 1% are summarized in Figure 6. The most abundant functions detected were related to chemoheterotrophy and aerobic chemoheterotrophy, which together accounted for 40–48% of the relative abundance. Significant increases from N0 to N1 were found for functions related to chemoheterotrophy, aerobic_chemoheterotrophy, nitrogen_fixation, phototrophy, photoautotrophy, cyanobacteria, oxygenic_photoautotrophy, and cellulolysis (Figure 6a and Table 3), while functions related to animal_parasites_or_symbionts, human_pathogens_pneumonia, iron_respiration, and all_human_pathogens were lower in N1 than in N0 (*p* < 0.05; Table 3). In addition, the functional groups related to C and N cycles were significantly affected by the nitrogen addition: the relative abundance of the C cycle functional group in N1 was significantly higher compared to N0, as was that of the N cycle functional group in N2 compared to N0 (*p* < 0.05 for both, Figure 6b).

### 3.5. Correlations among Soil Parameters with Soil Bacterial Community and Function

**Figure 7:**
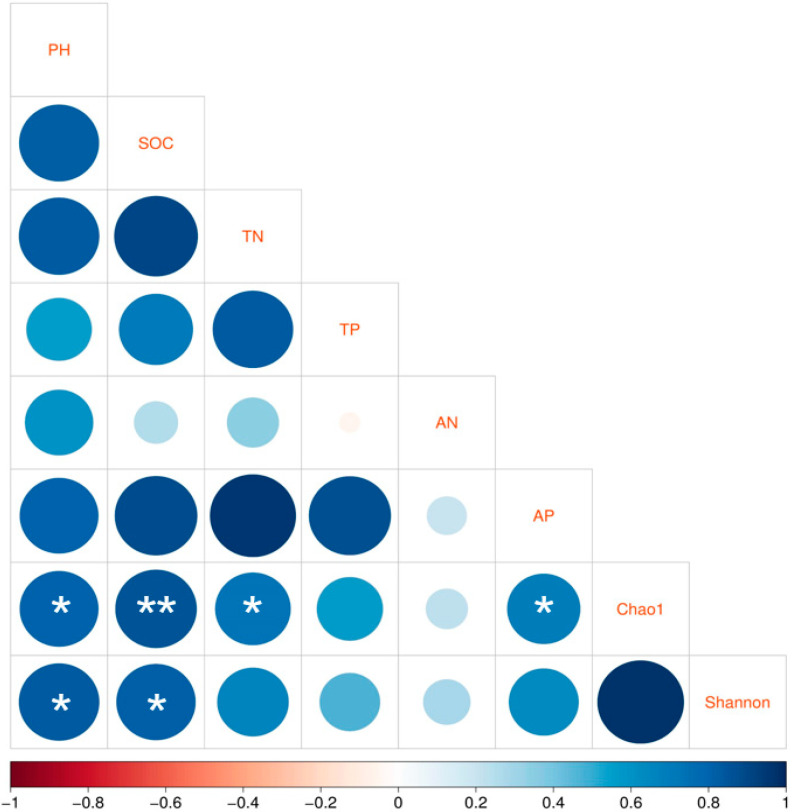
Pearson correlation analysis between the soil properties and the bacterial alpha diversity as expressed by Chao1 and Shannon indices. Correlations are indicated as * for *p* < 0.05 and ** for *p* < 0.01.

Pearson correlation analysis was applied to determine which correlations were present between soil bacterial α-diversity and the physicochemical factors of the soil. As illustrated in Figure 7, at a significance level of *p* < 0.05, the Chao1 diversity index correlated with pH, TN, and AP, and the Shannon index correlated with pH and SOC. The correlation between the Chao1 index and SOC was the strongest, with *p* < 0.01.

**Figure 8:**
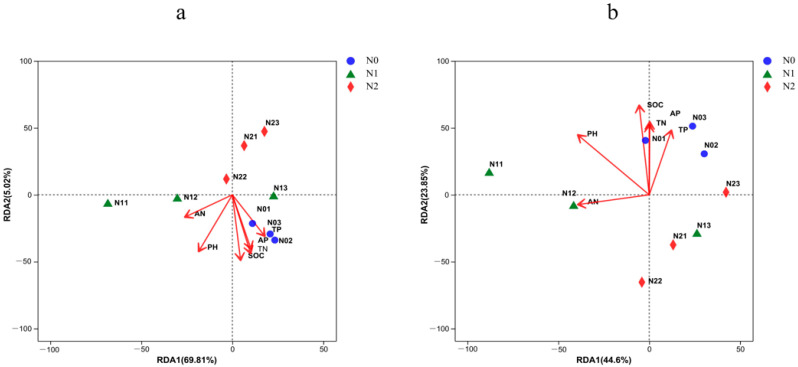
Redundancy analysis (RDA) of dominant soil bacteria phyla (**a**) and genera (**b**) constrained by the analyzed soil physicochemical properties. N0, 0 kg N·hm^−2^·a^−1^; N1, 40 kg N·hm^−2^·a^−1^; and N2, 80 kg N·hm^−2^·a^−1^.

Redundancy analysis was performed to identify the most important drivers behind the structural and compositional differences of the soil bacterial communities. At the phylum level (Figure 8a, Table 4), the cumulative variation in the first dimension of the RDA plot was 69.81%, and in the second dimension, it was 5.02%. Here, soil pH, SOC, and TN were the major factors explaining the phylum composition of the bacterial community, all indicating a negative correlation. At the genus level (Figure 8b, Table 4), the cumulative variations were 44.60% and 23.85% for the first and second dimension, respectively, and here, soil pH, SOC, and TN were the major positive factors explaining the composition of the soil bacterial communities.

**Figure 9:**
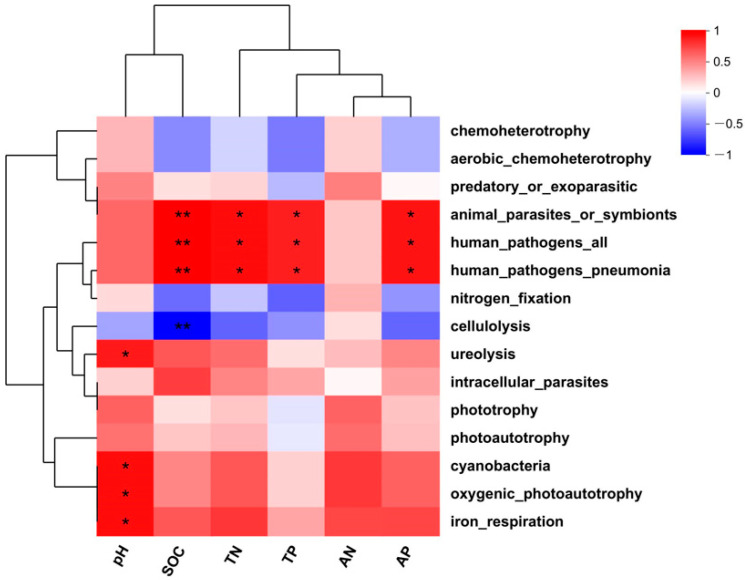
Correlation analysis between soil properties and soil bacterial functions. * for *p* < 0.05 and ** for *p* < 0.01.

The relationships of soil properties with the determined functions of the bacterial communities present in winter were visualized by means of a correlation heatmap (Figure 9). This illustrated that soil pH significantly and positively correlated with bacterial ureolysis, cyanobacteria, aerobic_chemoheterotrophy, and iron_respiration (*p* < 0.05). Strong (*p* < 0.01) positive correlations were identified between SOC animal_parasites_or_symbionts, human_pathogens_all, and human_pathogens_ pneumonia. These three bacterial functional groups also positively correlated with TN, TP, and AP at a level of *p* < 0.05. The only negative correlation that could be demonstrated was between SOC and bacterial functions related to cellulolysis (*p* < 0.01).

## 4. Discussion

### 4.1. Effects of Long-Term Nitrogen Deposition on Bacterial Diversity in Winter 

Nitrogen deposition can change the nutrient status of the soil substrate, causing changes in soil physicochemical indicators and affecting soil microorganisms [12,26]. In this study, we observed that the soil bacterial diversity determined in winter decreased with the increasing N deposition. A decade-long deposition of high N levels in the N2 treatment significantly reduced soil bacterial diversity compared to N0 that received no N, which is consistent with the published findings [27,28]. This reduction in diversity may be due to two effects. On the one hand, a long-term nitrogen deposition acidifies wetland soils, leading to an increase in acidophilic bacteria which may be accompanied with a decrease in other species and thus reduces soil bacterial diversity [29]. Previous studies have shown that bacterial community diversity is closely related to soil pH and soil acidification as a result of nitrogen deposition can significantly reduce soil bacterial diversity [30,31,32]. On the other hand, the decrease in microbial abundance may be attributed to an elevated N deposition leading to a decrease in subsurface C allocation [33,34]. Plants can provide soil microbes with assimilated carbon in exchange for other soil nutrients, including nitrogen [35]. However, when N is made available via N deposition, the amount of C released by plants may be less because less effort is required for the plants to acquire nitrogen [36]. Thus, as the N input increases, the carbon allocated in the subsurface pool decreases, reducing the use of carbon sources by microorganisms [37], thus reducing soil microbial diversity [38,39]. It seems these effects continue in winter during the dormant phase of most plants: we found that both the Shannon and Chao1 diversity indices of the bacteria present in the soil collected in winter significantly correlated with soil pH and with organic carbon content, validating these two possibilities. In addition, we found that the bacterial community diversity determined in winter was more sensitive to the nitrogen deposition compared to the growing season [34], which may be due to the lack of input from real-time aboveground plant carbon sources in winter, further highlighting the role of carbon limitation on soil bacterial diversity. This claim was also verified via a comparative analysis with the data from a previous study that found the effect of nitrogen deposition on soil bacteria in the growing season of the Three Rivers Plain wetlands [12]. This also suggests that the effect of nitrogen deposition on microbial diversity may be biased if only one season is investigated. To more accurately understand the microbial response to the nitrogen deposition, exploring the dynamic response of soil microbes to the nitrogen deposition under different seasons should be considered in future studies.

### 4.2. Effects of Nitrogen Deposition on Bacterial Compositions

Soil bacterial communities play a key role in regulating soil nutrients and as such, they affect plant growth; in turn, they are closely associated with the changes of soil properties caused by the nitrogen deposition [40,41]. We found that the dominant soil bacterial phyla in all three nitrogen deposition treatments were Proteobacteria and Acidobacteria. The latter phylum increased with the increasing nitrogen deposition; members of this phylum are typically more adaptable to acidic soils, and the long-term nitrogen deposition had led to soil acidification, providing a suitable environment for Acidobacteria, which is consistent with the previous results [5,30]. The relative abundance of less dominant phyla present above 1% also varied. The nitrogen addition significantly reduced the relative abundance of phyla-representing oligotrophic bacteria such as Myxococcota and Gemmatimonadota. However, great care should be taken when generalizing bacterial phyla to eutrophic or oligotrophic, as a wide range of physiological properties can exist between members of an individual microbial phylum. More detailed and accurate phylogenetic and taxonomic characteristics should be considered when documenting microbial community responses to infer microbial life strategies [42,43].

According to RDA analysis, soil pH, SOC, and TP were the main factors affecting soil bacterial community composition, which is consistent with the previous findings [12,15]. These results suggest that the N deposition regulates soil bacterial community composition in winter by changing the soil chemistry. However, our result found that soil TN and AN did not changed significantly (Table 1). This is probably due to the special wetland environmental characteristics that determines Sanjiang plain as a seasonal waterlogged wetland. The application N may be lost due to the surface water flow that was confirmed with our previous study [12]. Therefore, the soil bacterial community composition were mainly affected by SOC, soil pH, and TP. So, we think the research about the N deposition on wetland ecosystems should improve and avoid the waterlogged influence in the future. 

Furthermore, by regulating the relative abundance of specific taxa, the nitrogen deposition altered the microbial community composition [21,34]. Our results showed that the nitrogen addition significantly increased Candidatus_Solibacter and Bryobacter while decreasing the relative abundance of P3OB_42 and MND1. This may be mainly due to soil acidification brought about via nitrogen deposition. Members of the highly abundant genera of Candidatus_Solibacter and Bryobacter genera can be involved in the decomposition of organic matter, which they use as a carbon source. The increase in Candidatus_Solibacter and Bryobacter observed here with the nitrogen application treatment seemed to have promoted the complete decomposition of organic carbon, thus decreasing the soil organic carbon content, which is in line with other studies [21,31]. The correlation between different genera and soil chemistry in the three treatments indicated that the N addition could regulate the relative abundance of specific taxa of soil bacteria by changing the availability of soil nutrients.

### 4.3. Changes of Soil Bacterial Functions via the Nitrogen Deposition 

Microorganisms in wetland soils play an important role in the carbon and nitrogen balance; in elemental cycling, especially in C and N cycling; and in the energy flow [18,44,45]. Changes in microbial community composition also affect their functions, and this has been demonstrated to be an effect from the nitrogen deposition [26,46]. In this study, we identified particular bacterial functional groups that were associated with C and N cycling (chemoheterotrophy and aerobic_chemoheterotrophy, nitrogen_fixation) that had changed significantly with the increasing N deposition (Figure 7, Table 3) in support of the works that have demonstrated changes in the bacterial community structure that may affect elemental cycling in the soil [27,41]. In addition, with the increasing N deposition, functions related to phototrophy, oxygenic_ photoautotrophy, and photoautotrophy, as well as cyanobacteria and cellulolysis functional taxa, whose absolute abundance increased with the N deposition, may play key roles in the elemental cycle. In contrast, functions related to animal_parasites_or_symbionts, human_pathogens_pneumonia, iron_respiration, and human_pathogens_all functional groups were more abundant in untreated soil. Correlation analysis showed that the soil’s chemical properties regulated the functional structure of the bacterial communities. Therefore, the functions of the soil bacterial community dependent on the treatment and patterns were identified with the increasing nitrogen deposition due to the differences in soil properties.

## 5. Conclusions

When the soil of treatment plots in the Sanjiang Plain that had received a decade-long nitrogen deposition was analyzed in winter, it identified significantly reduced soil bacterial diversity as a result of high N deposition. Soil pH, SOC, and TN were the key factors on affecting the bacterial diversity and composition, while pH was the main factor regulating specific microbial communities involved in carbon cycling recognized by a functional group analysis of aerobic_chemoheterotrophy. Our study provides a basis for understanding the winter response of soil microbes to the nitrogen deposition and identified relationships between the soil microbial communities and soil physicochemical properties in the wetlands of the Sanjiang Plain.

## Figures and Tables

**Table 1:** Soil properties under different nitrogen concentration conditions.

N Deposition	pH	SOC (g·kg^−1^)	TN (g·kg^−1^)	AN (g·kg^−1^)	TP (g·kg^−1^)	AP (mg·kg^−1^)
N0	5.6 ± 0.1 a	46.5 ± 0.2 a	13.3 ± 0.2 a	0.85 ± 0.02 a	2.9 ± 0.1 a	45.3 ± 1.9 a
N1	5.4 ± 0.1 a	39.6 ± 0.2 b	12.9 ± 0.3 b	0.89 ± 0.02 a	2.7 ± 0.2 ab	42.1 ± 3.1 a
N2	4.7 ± 0.1 b	34.5 ± 0.1 c	12.2 ± 0.1 b	0.83 ± 0.02 b	2.6 ± 0.1 b	36.5 ± 1.5 b

Note: Results are reported as mean ± standard deviation (n = 3). Different letters within a row indicate significant differences (*p* < 0.05; one-way ANOVA) among the three treatments. AN: available nitrogen, AP: available phosphorous, SOC: soil organic carbon, TN: total nitrogen, TP: total phosphorus. N0: no N applied; N1: 40 kg N·hm^2^ per year for 10 years; N2: 80 kg N·hm^2^ per year for 10 years.

**Table 2:** Absolute abundances of the most abundant bacterial genera (top 10) under different nitrogen concentration conditions.

Genera	N0	N1	N2
*Candidatus_Solibacter*	3020.7 ± 161.4 b	3543.6 ± 317.5 a	3913.3 ± 243.9 a
*Haliangium*	2012.3 ± 161.8 a	2269.3 ± 465.2 a	1958.3 ± 265.66 a
*P3OB_42*	1596.1 ± 196.5 a	662.7 ± 193.5 c	1160.3 ± 126.8 b
*GOUTA6*	1077.3 ± 168.9 a	683.1 ± 73.2 a	1160.3 ± 126.7 a
*Ellin6067*	1086.3 ± 84.3 a	973.7 ± 95.4 a	1148.7 ± 121.2 a
*Bryobacter*	1057.7 ± 48.6 b	1252.1 ± 92.2 a	1232.1 ± 91.5 a
*Reyranella*	800.6 ± 73.4 a	1025.7 ± 86.1 a	809.1 ± 37.6 a
*Candidatus_Udaeobacter*	956.7 ± 81.8 ab	797.2 ± 59.2 b	1501.2 ± 136.1 a
*CL500_29_marine_group*	543.2 ± 35.7 ab	661.3 ± 76.2 a	381.3 ± 43.6 b
*MND1*	728.3 ± 41.4 a	630.3 ± 62.4 a	501.7 ± 40.1 b

Note: All results are reported as mean ± standard deviation (n = 3). Different letters within a row indicate significant differences (*p* < 0.05; ANOVA) among the three nitrogen deposition treatments tested in this study. N0, 0 kg N·hm^−2^·a^−1^; N1, 40 kg N·hm^−2^·a^−1^; N2, 80 kg N·hm^−2^·a^−1^.

**Table 3:** Absolute abundances of the most abundant (>1%) bacterial functional groups in the soil following the three treatments.

Functional Group	N0	N1	N2
*chemoheterotrophy*	3225.6 ± 103.1 b	5107.1 ± 169.8 a	3679.1 ± 150.3 ab
*aerobic_chemoheterotrophy*	3138.67 ± 178.8 b	5032.1 ± 182.4 a	3614.3 ± 172.5 ab
*predatory_or_exoparasitic*	2203.7 ± 147.6 a	2407.3 ± 101.9 a	2064.1 ± 116.4 a
*animal_parasites_or_symbionts*	1749.3 ± 64.8 a	1411.6 ± 139.7 b	1186.2 ± 107.5 b
*human_pathogens_all*	1735.6 ± 71.7 a	1389.3 ± 91.3 b	1155.7 ± 67.8 b
*human_pathogens_pneumonia*	1683.6 ± 60.4 a	1344.1 ± 91.4 b	1083.2 ± 85.2 c
*nitrogen_fixation*	670.3 ± 74.6 b	1093.7 ± 83.1 a	892.1 ± 49.6 ab
*ureolysis*	502.1 ± 42.7 a	590.3 ± 46.2 a	392.3 ± 23.6 a
*intracellular_parasites*	244.3 ± 20.8 a	182.7 ± 13.8 a	184.3 ± 14.1 a
*phototrophy*	209.1 ± 12.9 b	349.2 ± 21.8 a	213.3 ± 15.7 b
*photoautotrophy*	173.1 ± 10.7 b	289.2 ± 17.9 a	170.3 ± 11.5 b
*cyanobacteria*	148.3 ± 10.5 b	266.7 ± 20.6 a	112.3 ± 9.7 b
*oxygenic_photoautotrophy*	134.1 ± 10.9 b	235.2 ± 11.6 a	123.3 ± 10.2 b
*iron_respiration*	173.3 ± 13.5 a	221.3 ± 41.8 a	86.7 ± 6.3 b
*cellulolysis*	117.2 ± 3.5 b	188.6 ± 15.2 ab	229.3 ± 13.1 a

Note: Results are reported as mean ± standard deviation of triplicates. Different letters within a row indicate significant differences (*p* < 0.05; ANOVA) among the three treatments with N0, 0 kg N·hm^−2^·a^−1^; N1, 40 kg N·hm^−2^·a^−1^; N2, 80 kg N·hm^−2^·a^−1^.

**Table 4:** Mantel test determining the correlations between the environmental variables and bacterial community compositions.

	Phyla		Genera	
	*r* ^2^	*p*	*r* ^2^	*p*
pH	0.34	<0.05	0.41	<0.05
SOC	0.38	<0.05	0.43	<0.05
TN	0.18	>0.05	0.39	<0.05
TP	0.35	<0.05	0.27	>0.05
AN	0.16	>0.05	0.15	>0.05
AP	0.18	>0.05	0.21	>0.05

## Data Availability

Data are contained within the article.
